# Antiproliferative Activity of Hamigerone and Radicinol Isolated from *Bipolaris papendorfii*

**DOI:** 10.1155/2014/890904

**Published:** 2014-08-12

**Authors:** Periyasamy Giridharan, Shilpa A. Verekar, Akash R. Gohil, Prabhu Dutt Mishra, Amit Khanna, Sunil Kumar Deshmukh

**Affiliations:** Piramal Enterprises Ltd., 1 Nirlon Complex, Off Western Express Highway, Near NSE Complex, Goregaon (East), Mumbai 400 063, India

## Abstract

Secondary metabolites from fungi organisms have extensive past and present use in the treatment of many diseases and serve as compounds of interest both in their natural form and as templates for synthetic modification. Through high throughput screening (HTS) and bioassay-guided isolation, we isolated two bioactive compounds hamigerone (1) and radicinol (2). These compounds were isolated from fungus *Bipolaris papendorfii*, isolated from the rice fields of Dera, Himachal Pradesh, India. The structures of the compounds were established on the basis of spectroscopic data, namely, NMR (^1^H, ^13^C, mass, and UV). Both compounds were found to be antiproliferative against different cancer cells. Furthermore we have also noted that both compounds showed increase in apoptosis by favorably modulating both tumor suppressor protein (p53) and antiapoptic protein (BCL-2), and in turn increase caspase-3 expression in cancer cells. This is the first report of these compounds from fungus *Bipolaris papendorfii* and their anticancer activity.

## 1. Introduction

Microfungi are a rich source of chemical diversity [1–3], and its metabolites are used by the pharmaceutical industry either in the native form or as derivatives [4–6]. As only a small part of the Mycota is known and most fungi produce several unknown metabolites, fungi are still one of the most promising sources for new lead compounds. Hence fungi are the objective in numerous high throughput screening (HTS) programs targeting new pharmaceutics and other bioactive components [7–10].

In our ongoing pharmacological screening program on biodiversity of fungi present in Indian landscape using high throughput screening (HTS), we have identified a notable antiproliferative activity against cancer cells in extracts/fractions of a fungus* Bipolaris papendorfii*, coded as PM0853873 isolated from the rice fields of Dera, Himachal Pradesh, India. Hamigerone was originally reported as the major secondary metabolite of* Hamigera avellanea* but no pharmacological activity was reported [11]; similarly radicinol was isolated from* Cochliobolus lunata* but no pharmacological activity was reported [12]. Apoptosis is essential in the homeostasis of normal tissues of the body. The two important genes involved in this process are the BCL-2 gene and p53 gene. BCL-2 is a gene of antiapoptosis and is crucial in preventing apoptosis whereas p53 gene is known as tumor suppressor gene. Cellular stresses, particularly DNA damage, are sensed by proteins such as ATM and DNA-PK, which phosphorylate and stabilize p53 which in turn cause apoptosis to the cancer cells [13]. The apoptotic pathway is related to induction of p53 and this pathway is held in check by the antiapoptotic gene BCL-2 [14]. The protooncogene BAX forms a heterodimer with BCL-2 and accelerates the process of apoptosis. We describe here the identification and bioactivity of the lead compounds hamigerone and radicinol from this culture. In this study, we also demonstrate for the first time the novel* in vitro* antiproliferative properties of hamigerone and radicinol in cancer cells. Both the compounds are inducing apoptosis in Panc-1 cancer cells by favorably modulating p53, BCL-2, and caspase 3 expression.

## 2. Materials and Methods 

### 2.1. General

HPLC was performed in Lichrosphere RP-18, 125 × 4 mm column using Shimadzu LC-2010 CHT Liquid Chromatography. Water preparative HPLC was used for final purification. Solvents used were of HPLC grade and normal column chromatography (CC) was performed with distilled commercial-grade solvents. Silica gel (SiO_2_, 200–300 mesh) was used for CC and GF_254 _(30–40 mm). TLCs were procured from Merck. NMR spectra in CDCl_3_ were recorded on Bruker 300 MHz spectrometer with TMS as the internal standard with chemical shift *δ* values in ppm and coupling constant J in Hz. ESI LC-MS was from Bruker Daltonics. Flash chromatography was performed on CombiFlash Sq 16X Teledyne Technologies Company ISCO attached with UV/VIS detector, (RediSep Flash Column silica 12 g, CHCl3 / MeOH gradient system). Final purification was achieved by preparative HPLC using RP-18 column (10X25 mm, 10 *μ*) and acetonitrile/water as a solvent system.

### 2.2. Cell Culture Reagents

All the cell lines were obtained from ATTC, USA. McCoy's 5a medium, MEM, RPMI-1640, FBS, BSA, isopropanol, dimethylsulfoxide (DMSO), formaldehyde, 3-(4,5-dimethylthiazol-2-yl)-2,5-diphenyl tetrazolium bromide, Triton X-100, and PBS were obtained from Sigma, USA. Hoechst 3342 and Dylight 549 were obtained from Thermo Fisher Scientific, USA. All the antibodies, namely, BCL-2 (SC-738), p53 (SC-126), and cleaved caspase 3 (SC-22171), were procured from Santa Cruz Biotechnology, Inc., USA.

### 2.3. Isolation and Identification of Fungus

**Figure 1:**
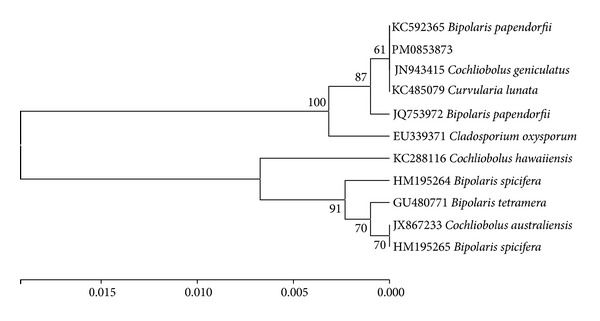
Phylogenetic analysis of the ITS region sequence obtained from sample PM0853873 in comparison with the nearest type strain sequences. The tree was constructed based on rRNA gene sequences (ITS region) using the Maximum Composite Likelihood Method.

The strain PM0853873 was isolated from the soil samples collected from Dera rice field during October 2007. The culture was isolated using soil plate method [15] using potato carrot agar (Hi Media) supplemented with 50 mg/L of chloramphenicol and maintained on potato dextrose agar (Hi Media) supplemented with 50 mg/L of chloramphenicol for identification and fermentation. The strain was identified as* Bipolaris papendorfii* by partial sequence analysis of the internal transcribed spacer region (ITS) using ITS1 and ITS4 primers [16]. A nucleotide to nucleotide BLAST [17] query of the gene bank database (http://www.ncbi.nlm.nih.gov/BLAST) recovered KC592365* Bipolaris papendorfii* (92%) as the closest match to the ITS rDNA of PM0853873 (100.0%). Evolutionary analyses (Figure 1) were performed using MEGA6 [18].

### 2.4. Large-Scale Production of the Fungus

A loop full of the well grown culture from slant maintained on potato dextrose agar (PDA) was transferred to a 500 mL conical flask with 100 mL liquid medium containing soluble starch 1.5 g; soya bean meal 1.5 g; yeast extract 0.2 g; corn steep liquor 0.1 g; glucose 0.5 g; CaCO_3_ 0.2 g; NaCl 0.5 g; glycerol 1.0 g in demineralized water at pH 5.5. This was grown on rotary shaker at 220 rpm for 72 h at 28°C and was used as seed medium. Potato dextrose broth medium (Hi Media) was used for production. The pH of the medium was adjusted to 6.5 prior to sterilization. Twenty-five, 1000 mL flasks containing 200 mL of the above medium were inoculated with 1% of the seed culture and incubated on rotary shaker at 220 rpm for 72 h at 28°C.

### 2.5. Purification of Compounds

5 L fermentation broth was filtered through Whatman number 1 filter paper to separate biomass. Methanol was added to the biomass, stirred for 40 min, and filtered. The filtrate was evaporated on rotary evaporator to remove methanol and aqueous residue was partitioned with ethyl acetate; the organic layer was concentrated on rotary evaporator to remove solvent, which yielded 500 mg crude extract. The crude extract was subjected to flash chromatography 16X on ISCO Combiflash system using RediSep silica column (SiO_2_, 200–300 mesh, 12 g; petroleum ether/ethyl acetate gradient). Semipure compounds** 1** and** 2** were obtained from the fractions eluted with 10% ethyl acetate in petroleum ether. Final purification of compound** 1** and** 2 **was performed on preparative HPLC (Waters PrepLC 4000 system, RP-18 column 8 × 250 mm (Lichrosphere), 10 *μ*; 2–100% acetonitrile in 30 min gradient against water, 5 mL/min flow). The peak at RT 28.2 was collected and evaporated the solvent to get 8 mg pure compound** 1**. In a similar preparative HPLC experiment semipure compound** 2** appeared at 16 min to give 12 mg pure compound. The purity of the compounds were determined by Analytical HPLC.

### 2.6. Identification of the Substances

These compounds were characterized by spectroscopic data (UV, ^1^H-NMR, ^13^C-NMR, and LC-MS). Compounds** 1** and** 2** were identified as hamigerone and radicinol respectively. LC MS data indicated the molecular weight of compound** 1** as 412 and compound** 2** as 238.

## 3. Screening for Anticancer Activity

### 3.1. Cell Proliferation Assay

Cell growth was measured by 3-(4,5-dimethylthiazol-2-yl)-2,5-diphenyl tetrazolium bromide (MTT) method [19]. Cells were seeded at the appropriate concentrations to prevent confluence throughout the experiment. After 24 h of incubation, cells were treated with serial concentrations of the compounds. Control cells were treated with equal concentration of DMSO (never exceeding 0.1%). At 72 h after treatment, aliquots of 10 *μ*L of MTT (5 mg/mL) were added to each well and incubated for 4 h at 37°C. The supernatant was removed and 100 *μ*L of isopropanol was added. The color intensity of reduced MTT was measured using Tecan Sapphire multifluorescence microplate reader (Tecan, Germany, GmbH) at 595 nm. DMSO-treated cells were considered untreated control and assigned a value of 100%.

### 3.2. *In Vitro* Protein Expression (by High Content Screening)

Panc-1 cells were seeded in 96-well plates at a density of 1 × 10^4^ cells/well. 24 h after seeding, medium was replaced with fresh medium. Panc-1 cells were treated with 1.9 *μ*M of hamigerone and 10.50 *μ*M of radicinol for 12 h. At the end of every time point, to determine the protein expression, the cells were fixed with 3.7% formaldehyde in PBS for 10 minutes at room temperature, followed by permeabilization with 0.15% Triton X-100 for 10 minutes. After permeabilization, the cells were blocked with 5% BSA for 2 h. After blocking step specific primary antibody was added for 1 h. Following primary antibody incubation, the nucleus was stained with Hoechst 3342 (blue) and primary antibodies of different protein (BCL-2, p53, and caspase 3) were localized by secondary antibody labeled with Dylight 549 (red). Immunofluorescence of BCL-2, p53, and cleaved caspase 3 was determined by scanning the plates on Cellomics Array Scan VTI HCS Reader with 20X magnification (Thermo Fisher Scientific Inc., Waltham, MA).

All the data points were analyzed using the target activation and molecular translocation bioalgorithm of Cellomics and the quantitative data was expressed as percentage (%) activation in comparison to the DMSO control cells. 1000 cells were counted for each replicate well and the results were presented as an average ± SE. Bioapplication enables identification of the cell and cytoplasmic areas; the blue traces define the cell boundaries, while the orange traces cells rejected from the analysis. The scoring outputs used were total cellular/nuclear intensity and redistribution of fluorescence intensity from the nucleus to the cytoplasm (entropy intensity change) [20, 21].

### 3.3. Statistical Analysis

The data shown are the mean values of at least three replicate experiments and expressed as means ± SD. Differences between groups were analyzed using two sided *t*-test and ANOVA with *P* < 0.05 considered statistically significant. In cases in which averages were normalized to controls, the SDs of each nominator and denominator were taken into account in calculating the final SD. Statistical analyses were conducted using GraphPad Prism software 3.03 package (GraphPad Software, Inc., CA, USA).

## 4. Results and Discussion

### 4.1. Isolation and Structural Elucidation

**Figure 2:**
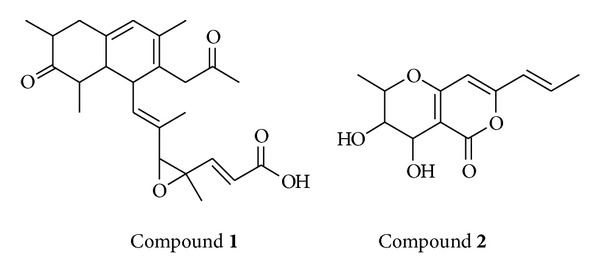
Structures of hamigerone (**1**) and radicinol (**2**).

The bioactivity guided purification of the crude extract obtained from the fermented whole broth yielded pure bioactive compounds** 1** (hamigerone) and** 2** (radicinol) (Figure 2). These compounds were characterized by spectroscopic data (UV, ^1^H-NMR, ^13^C-NMR, and LC-MS). All the values were in complete accord with that of the reported values [11, 12, 22].

Hamigerone was previously isolated from the fungus* Hamigera avellanea* and exhibits* in vitro* growth inhibitory activity against phytopathogenic fungi [11].

Radicinol, the phytotoxic compound, was produced on carrot disks by* Cochliobolus lunata* IFO 6288 [12]. It was also produced by* Bipolaris coicis*,* Alternaria radicina,* and* A. chrysanthemi* [22–24].

### 4.2. Antiproliferative Effect and* In Vitro* Protein Expression of Hamigerone and Radicinol

Hamigerone induced cytotoxicity in various cancer cell lines with an IC_50_ ranging from 1.9 *μ*M to 4.3 *μ*M. Similarly radicinol showed lesser efficacy as a poor inducer of cytotoxicity towards the cancer cell lines with an IC_50_ in the range of 10.50 *μ*M–25 *μ*M (Table 1). Both the compounds were not toxic to normal breast epithelial cell line (MCF10A), as there was no growth inhibition up to a concentration of 30 *μ*M. These findings suggest that these compounds are specific towards the abnormally proliferating cells [25] wherein hamigerone showed better toxicity to cancer cells as compared to radicinol. Based on the potent antiproliferation activity of both hamigerone and radicinol in Panc-1 cells, these compounds were further profiled for p53, BCL-2, and caspase 3 activity using high content screening tool Cellomics VTI Array Scan.

**Figure 3:**
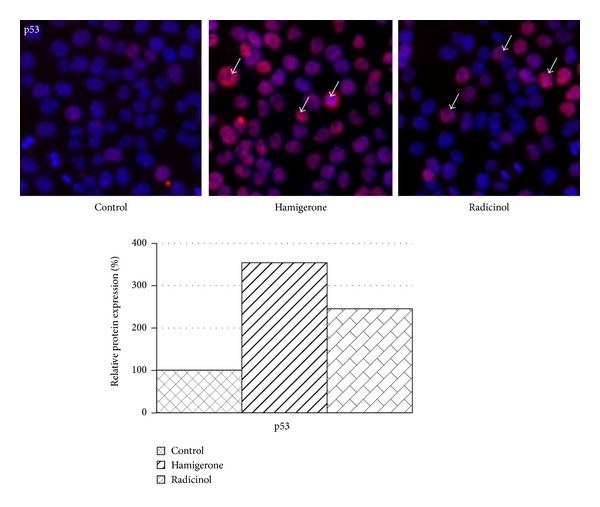
Elevation of p53 expression on treatment with hamigerone and radicinol in Panc-1 cells. The nucleus was stained with Hoechst 3342 (blue color), the expression of p53 protein levels was detected using Dylight 549 (red color), and the represented images are composite images of both channels. The quantitative data of p53 expression against untreated control cells is denoted as bar diagram. 1000 cells were counted for each replicate well and the results were presented as an average ± SE.

The p53 protein is known as a cellular gate-keeper. In general, in more than 70% of the tumors p53 status is either mutated or deleted. Activating p53 in cancer cells aids in driving the cancer cells into apoptosis. We have analyzed p53 protein expression in Panc-1 cells after 12 h of treatment with hamigerone and radicinol. The results showed 3.5-fold and 2.4- fold increased expression of p53 in Panc-1 cells (Figure 3), respectively. The quantitative expression level of this protein is represented as bar diagram.

It was also observed that hamigerone showed more significant induction of p53 (red color Ch2) when compared with radicinol. The significance of p53 in cell cycle arrest was also noted with* Selaginella tamariscina* extract in human ovarian cancer cell line A-2780 [26].

**Figure 4:**
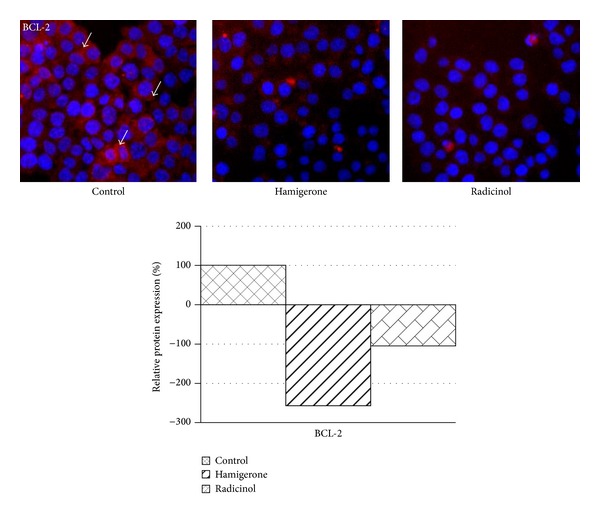
Decreased expression of BCL-2 levels on treatment with hamigerone and radicinol in Panc-1 cells. The nucleus was stained with Hoechst 3342 (blue color), the expression of BCL-2 protein levels was detected using Dylight 549 (red color), and the represented images are composite images of both channels. The quantitative data of BCL-2 expression against untreated control cells is represented as bar diagram. 1000 cells were counted for each replicate well and the results were presented as an average ± SE.

BCL-2 is an antiapoptotic protein. On treatment with hamigerone we noted 2.5-fold decreased expression of BCL-2 as compared with untreated control cells (Figure 4), while radicinol showed marginal reduction (1-fold). This data reveals that hamigerone was more potent than radicinol in reducing BCL-2 expression in Panc-1 cells. The bar diagram indicates the quantitative expression of BCL-2 protein levels. In another study resveratrol, a phytoalexin present in grapes and other food products, induces apoptosis in cancer cells by decreasing BCL-2 expression in HL-60 cells and gives rise to proteolytic cleavage of caspase substrate PARP [27] thereby causing apoptosis to the cancer cells.

**Figure 5:**
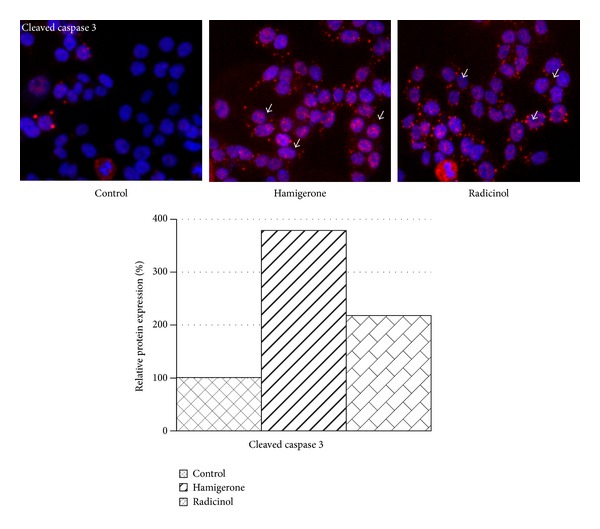
Induction of caspase 3 expression on treatment with hamigerone and radicinol in Panc-1 cells. The nucleus was stained with Hoechst 3342 (blue color), the expression of caspase 3 protein levels was detected using Dylight 549 (red color), and the represented images are composite images of both channels. The quantitative data of caspase 3 expression against control is showed as bar diagram. 1000 cells were counted for each replicate well and the results were presented as an average ± SE.

Furthermore we evaluated the cleaved caspase 3 activity in the cells treated with these compounds. In Panc-1 cells cleaved caspase 3 levels were higher (3.9-fold and 2.1-fold) in cells treated with hamigerone and radicinol as compared to untreated cells, respectively (Figure 5). These findings suggest the cytotoxicity effect of hamigerone and radicinol in cancer cells could be via induction of caspase 3 activation. The quantitative expression level of this protein against untreated controls is represented as bar diagram.

Of all the proteins involved in the activation and execution of apoptosis, the caspase 3 stands out as being crucial for this process [28].

In this study we have identified that hamigerone and radicinol interact with multiple cancer targets whose implication in cancer drug discovery is well described previously. Based on our findings, these molecules can become a potential start-up point for developing new scaffold which can modulate potential cancer targets.

## Figures and Tables

**Table 1:** IC_50_ of hamigerone and radicinol in different cell lines.

Cell line	IC_50_ in *µ*M (±SD)			
	Hamigerone	Radicinol	Gemcitabine	Flavopiridol
Panc-1 (pancreatic)	1.9 ± 0.92	10.50 ± 1.4	0.38 ± 0.04	0.62 ± 0.05
ACHN (renal)	4.3 ± 0.78	14.28 ± 1.8	0.78 ± 0.07	0.91 ± 0.11
Calu1 (lung)	2.91 ± 1.1	12.60 ± 0.87	0.98 ± 0.11	0.79 ± 0.19
H460 (non-small cell lung)	2.42 ± 0.63	21.6 ± 3.2	1.23 ± 0.23	0.82 ± 0.09
HCT116 (colon)	2.91 ± 0.87	25 ± 2.9	1.15 ± 0.21	0.73 ± 0.08
MCF10A (normal breast epithelium)	>30	>30	>10	>10

IC_50_ values of hamigerone and radicinol in different cancer cells at 48 h. The cells were exposed to different concentrations at end of 48 h; cytotoxicity was measured using live cell dehydrogenase assay.
